# Systems analysis reveals differential expression of endocervical genes in African women randomized to DMPA-IM, LNG implant or cu-IUD

**DOI:** 10.1016/j.clim.2023.109750

**Published:** 2023-10

**Authors:** Prachi Mehrotra Gupta, Christina Balle, Gregory K. Tharp, Sydney A. Nelson, Melanie A. Gasper, Bryan Brown, Arghavan Alisoltani, Maricianah Onono, Thesla Palanee-Phillips, Gonsagrie Nair, Hosseana Ayele, Laura Noel-Romas, Jo-Ann S. Passmore, Adam D. Burgener, Renee Heffron, Heather B. Jaspan, Steven E. Bosinger

**Affiliations:** a Emory National Primate Research Center (ENPRC) Genomics Core Laboratory, Division of Microbiology & Immunology, Emory University, Atlanta, GA, USA; b Department of Pathology, Institute of Infectious Disease and Molecular Medicine, University of Cape Town, South Africa; c Seattle Children's Research Institute, Seattle, WA, USA; d Department of Pediatrics, University of Washington, Seattle, WA, USA; e Department of Microbiology-Immunology, Northwestern University Feinberg School of Medicine, Chicago, IL, USA; f Kenya Medical Research Institute, Kisumu, Kenya; g Wits RHI, University of the Witwatersrand, Faculty of Health Sciences, School of Public Health, Johannesburg, South Africa; h Desmond Tutu HIV Centre, Cape Town, South Africa; i Department of Pathology, Case Western Reserve University School of Medicine, Cleveland, OH, USA; j National Health Laboratory Service, Cape Town, South Africa; k CAPRISA DSI-NRF Centre of Excellence in HIV Prevention, University of Cape Town, South Africa; l Department of Epidemiology, School of Public Health, University of Washington, Seattle, WA, USA; m Department of Global Health, University of Washington, Seattle, WA, USA; n Emory University School of Medicine, Department of Pathology & Laboratory Medicine, GA, USA; o Emory Vaccine Center, Emory University, GA, USA

**Keywords:** Female genital tract, Contraception, Transcriptome, Reproductive health, Randomized clinical trial, Mucosa

## Abstract

Although effective contraceptives are crucial for preventing unintended pregnancies, evidence suggests that their use may perturb the female genital tract (FGT). A comparative analysis of the effects of the most common contraceptives on the FGT have not been evaluated in a randomized clinical trial setting. Here, we evaluated the effect of three long-acting contraceptive methods: depot medroxyprogesterone acetate(DMPA-IM), levonorgestrel(LNG) implant, and a copper intrauterine device (Cu-IUD), on the endocervical host transcriptome in 188 women from the Evidence for Contraceptive Options and HIV Outcomes Trial (ECHO) trial. Cu-IUD usage showed the most extensive transcriptomic changes, and was associated with inflammatory and anti-viral host responses. DMPA-IM usage was enriched for pathways associated with T cell responses. LNG implant had the mildest effect on endocervical gene expression, and was associated with growth factor signaling. These data provide a mechanistic basis for the diverse influence that varying contraceptives have on the FGT.

## Highlights

• Contraceptives significantly impact the immune system of the female genital tract (FGT)
• The impact of DMPA, LNG implant, and Cu-IUD on the FGT was studied in 188 women from the ECHO trial using functional genomics
• Cu-IUD led to elevated metallothionein responses associated with increased inflammation
• DMPA usage was associated with enhanced T cell recruitment and signaling
• LNG implant had the mildest impact on the FGT environment

## Graphical abstract

**Figure:**
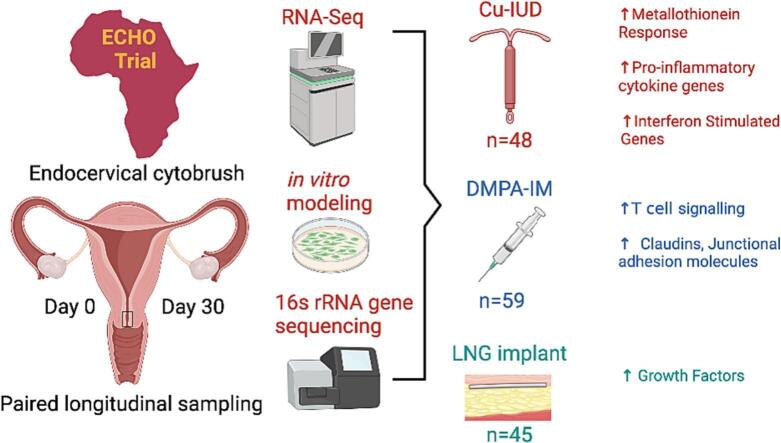

## 1 Introduction

Young women in sub-Saharan Africa are at a high risk of unintended pregnancies [1,2], which are associated with high maternal and infant mortality, and morbidity, especially in developing countries [3]. Non-barrier contraceptive (NBC) methods, including hormonal contraceptives (HC) have become an important tool in preventing unintended pregnancies and associated sequelae. In recent years, several studies have indicated that HCs can alter the female genital tract (FGT) mucosal environment in a variety of ways, including recruitment of HIV target cells [4,5], influencing changes in microbiota [[6], [7], [8], [9]] and instigating inflammation [[10], [11], [12]], with the potential to increase the risk of sexually transmitted infections (STIs), including HIV [[13], [14], [15], [16]]. Recently, however, the Evidence for Contraceptive Options and HIV Outcomes (ECHO) trial, a randomized open-label trial of three highly-effective long-acting contraceptives, concluded that there is no significant difference in HIV incidence among women randomized to intramuscular depot medroxyprogesterone acetate (DMPA-IM), copper T intrauterine device (Cu-IUD) and levonorgestrel (LNG)-implant [17]. Evidence has suggested however that the effect of injectable HC use on HIV acquisition may be modified by the composition of the vaginal microbiota [18] with different HIV risk profiles observed among DMPA-IM users with a *Lactobacillus*-dominated versus *Lactobacillus*-depleted vaginal microbiota [19]. Recent studies have indicated that DMPA-IM can alter host gene expression associated with mucosal epithelial barrier integrity by reducing the levels of epithelial growth factors [[20], [21], [22], [23], [24]], epithelial repair, cell junction and maintenance proteins [[25], [26], [27]], and protease inhibitors, including matrix metalloproteinase (MMPs) tissue inhibitors (TIMPs) [20,25,28,29] in the FGT. Furthermore, in non-human primates (NHPs), DMPA (depo-medroxyprogesterone acetate) treatment has led to reduced genital levels of the cell-cell adhesion molecules, weakened epithelial barrier function [30,31] and have been associated with vaginal atrophy [[32], [33], [34]]. However, thinning of the vaginal epithelium in humans using DMPA-IM has not been demonstrated at the dose administered [7,8,[35], [36], [37], [38]]. Until recently, most of the data linking NBC use to changes in the mucosal environment have been derived from observational studies, which are potentially biased by the behavioural confounders that may mask the true biological effects [39,40]. To allow for a better understanding of the biological effects of DMPA-IM, LNG-implant and Cu-IUD initiation on the FGT host environment, we conducted a nested mucosal sub-study of women participating in the ECHO trial (R01HD089831), to evaluate endocervical host transcriptomics immediately prior to and after one month of contraception initiation with ongoing use.

## 2 Methods

### 2.1 Study design

Within the ECHO trial (NCT02550067), which assessed the HIV-1 incidence among women randomized to Cu-IUD, LNG implant, or DMPA-IM, this nested sub-study aimed to evaluate the impact of these contraceptives on the endocervical transcriptome. In brief, the ECHO trial enrolled non-pregnant, HIV-seronegative women between 16 and 35 years of age who were sexually active, desiring effective contraception and willing to be randomized to contraception between December 2015 and September 2017^17^. Women were excluded if they reported having used injectable contraception, an implant or IUD in the last 6 months, had previously had a hysterectomy or sterilization, and/or had untreated gonorrhea or chlamydia. Ethical approvals for this nested sub-study were obtained from the Research Ethics Committees at the University of Cape Town (HREC 371/2015), University of the Witwatersrand (HREC PRC 141112), KEMRI (SERU/CMR/P0014/3109), University of Washington (STUDY00000261), and FHI360 (523201). All participants provided written informed consent prior to participating in the study, including consent for storage and processing of specimens at international laboratories. Following ethics approvals, a total of 1359 women were enrolled in this a priori sub-study, (Wits RHI, 113 women; Emavundleni, 469 women; KEMRI, 777 women). Of these, 188 had matched endocervical samples collected at baseline and one-month post-contraception initiation available for RNA-Seq analysis.

### 2.2 STI and BV testing

Women were tested for *C. trachomatis* (CT) and *N. gonorrhoeae* (NG) at baseline using endocervical swabs and treatment was provided upon etiologic diagnosis or when a woman presented with symptoms, according to national guidelines. For CT/NG testing, GeneXpert Instrument Systems platform (Cepheid Inc., US) with the Abbott Real Time PCR assay (Abbott Molecular, US) were used at Wits RHI and Emavundleni sites, while the Panther System (Hologic Inc., US) was used at the KEMRI site [17]. Nugent scoring (BV negative [Nugent 0–3], intermediate [Nugent 4–6], or positive [Nugent 7–10]) was conducted by the National Institute for Communicable Diseases (NICD) laboratory in Johannesburg, South Africa.

### 2.3 RNA-Seq library preparation and quality control analysis of data

Cervical cytobrush samples collected at baseline and at one-month follow-up visit from 188 matched participants (*n* = 376) were processed for RNA sequencing of cervical cells (Supplementary Fig. 1A) as described previously [41], with 6 h as the window of delivery from clinic to the lab. Samples were defined as suitable quality based on the following criteria: (i) >5 M unique reads mapping to the host, (ii) a median coefficient of variation (CV) coverage ≤1.4, (iii) a RIN score ≥ 2 and (iv) variance in relative log expression (RLE) between −3.5 and 3.5 (Supplementary Fig. 2).

### 2.4 Differential expression analysis

A paired differential expression analysis, i.e. post-vs-pre (one-month vs. baseline) comparison, was carried out for each study arm. For the paired analyses, only protein-coding probe-target genes were retained: hg19 probe coordinates from the Illumina RNA Exome kit were cross-mapped to hg38 genome assembly using cross-map, an assembly converter tool. Genes which are classified as ‘protein-coding’ by the gene biotype field in the hg38 GTF file and for which the exon coordinates showed an overlap of at least one base with any of the probe coordinates, were included in the set of protein-coding probe-target genes (19,543 genes). The overlap across the exon coordinates and probe coordinates was computed using the ‘closest’ tool from bedtools. DESeq2 (version 1.22.1) in Bioconductor/R platform was used to perform the differential expression analysis within the study arms, with a single-factor experimental design formula, design = ∼assigned_contraceptive [42]. The assigned contraceptive factor includes six levels, DMPA-IM, LNG implant and Cu-IUD at baseline and at one month. Power calculations were run with the PROPER package [43] using metrics described previously [41]. Differential expression analysis results were extracted for each post-vs-pre comparison. The following criteria defined DEGs within each comparison: false discovery rate (FDR) <0.05, absolute fold change (FC) >2, i.e. absolute log_2_FC > 1, a standard error estimate for the log_2_fold-change estimate (lfcSE) <1 and mean expression of a gene >10. GSEA was performed on the regularized log expression data using the GSEA tool from MSigDB [44]. The enrichment analysis was carried out for post-vs-pre comparisons for the following pathway collections: Hallmark [45], Kyoto Encyclopedia for Genes and Genomes (KEGG) [46], PID [47], Reactome [48] and BioCarta [49], and custom in-house gene-sets that include Serpins and Integrins [32,[50], [51], [52], [53], [54]]. Heatmaps were generated using the ComplexHeatmap package in R. The regularized log expression for each gene was normalized by the mean expression across baseline samples and plotted based on a gradient color-scale.

### 2.5 Defining Lactobacillus-dominant versus Lactobacillus-depleted vaginal microbiota at baseline in DMPA-IM study arm

To investigate if the cervicovaginal transcriptomic changes observed in women using DMPA-IM were associated with a *Lactobacillus*-rich microbiota, subjects were defined as either *Lactobacillus*-dominant (LD) or *Lactobacillus*-depleted (nonLD) at baseline, prior to contraception initiation. LD/nonLD classification was determined from 16S rRNA microbiome sequencing, described in our prior dataset [55]. Community state types of vaginal microbiomes obtained were defined as: LD (CST I—B and CST III-A); nonLD (CST IVs [IV-A, IV-B, IV-D]).

### 2.6 Deconvolution of immune cell types using RNA-seq data of women assigned to cu-IUD

The normalized gene counts of samples in Cu-IUD study arm (*n* = 48), collected at baseline and at one month post Cu-IUD initiation were processed with CIBERSORTx web-server [56] (https://cibersortx.stanford.edu/) against its default LM22 gene signature matrix, with 1000 permutations, using both relative and absolute modes. To validate the results of CIBERSORTx, the data was also processed using xCell webserver [57] (https://xcell.ucsf.edu/) against its default 64 signature cell types. Of the 64 signature cell types, 34 were immune cell types that were median centered for downstream analysis. The following immune cell types were taken to be significantly differentially abundant: (i) immune cells types with *p*-value ≤0.05 in both CIBERSORTx relative and absolute modes, (ii) immune cells types with FDR adjusted p-value (q-values) ≤0.05 in at least one of the CIBERSORTx modes, (iii) immune cell types for which the direction of change in their proportion was consistent across results from absolute and relative modes in CIBERSORTx and xCell algorithms. Heatmaps were generated using the ComplexHeatmap package in R [58] for results obtained from the relative and absolute modes in CIBERSORTx. The optimum number of clusters for hierarchical followed by K-means clustering of all women according to the proportion of immune cell types was determined using NbClust R package [59].

### 2.7 Cell culture and luminex

Human Endocervical Epithelial cells (END1/E6E7) were purchased from the American Type Culture Collection (ATCC; Manassas, VA, USA) and cultured in keratinocyte serum-free medium (KSFM; Gibco Life Technologies, Grand Island, NY, USA) with 0.1 ng/ml human epidermal growth factor, 0.05 mg/ml bovine pituitary extract, 0.4 mM CaCl_2_, 100 units/ml penicillin, and 100 μg/ml streptomycin (Gibco Life Technologies). Following enumeration, 150,000 cells were plated in wells of a 24-well plate. Upon reaching ∼90% confluency, cells were treated for 24 h with dilutions of copper chloride (Sigma) that correspond to 9 × 10^−10^, 9 × 10^−8^, 9 × 10^−6^, 9 × 10^−4^ g/ml of Cu^2+^. Initial CuCl_2_ solutions were prepared [60] and diluted further in KSFM for these experiments. Heat-killed *E. coli* (HKEC; 2 × 107 colony forming units/ml) was added to cells for the positive control, and media alone was used for the negative control. After 24 h of exposure to copper, HKEC, or media, supernatants were collected and stored at −80 °C. Cell monolayers were lysed in RLT (Qiagen) with β-mercaptoethanol (Sigma) and stored at −80 °C until use.

Supernatants were assessed for the concentrations of IL-1b, IL-6, IP-10 and VEGF via a BioRad Bioplex Pro custom kit as per the manufacturer's protocol. All data were acquired on a Luminex 200 instrument and analyzed within the BioPlex Manager Software, where a 5 Parameter Logistic regression formula was used to interpolate unknown cytokine concentrations from standard curves. Student's *t*-test was run for the mean cytokine concentration in each condition, i.e. mean of 3 experimental replicates, versus the negative control (media only). Correction for multiple comparisons was performed following the method of Benjamini and Yekutieli [61]. Data visualization and statistical analysis was performed in R (v4.1.2).

### 2.8 Statistical analysis

All downstream statistical analyses were performed in RStudio. Differences in study population characteristics according to study arm were tested using Pearson's Chi-squared test or Fisher's exact test (when the expected value was <5) for count data and unpaired Student's *t*-test for differences in mean (parametric data) and unpaired Mann-Whitney *U* test for differences in medians (non-parametric data) with post-hoc testing. The abundances of immune cell types followed sparse and non-normal distributions (Shapiro-Wilk test), hence for the immune cell deconvolution analysis, Wilcoxon–Mann–Whitney test was used to compare the population of immune cell types at baseline and one month post Cu-IUD initiation.

## 3 Results

### 3.1 Cohort characteristics

For this nested mucosal sub-study of the ECHO trial, we included a total of 188 women with matched endocervical cytobrush samples obtained prior to, and one month after contraceptive initiation (DMPA-IM, *n* = 66; LNG-implant, *n* = 61; Cu-IUD, n = 61). Subjects were enrolled from the Wits RHI (Johannesburg; South Africa), Desmond Tutu Health Foundation (DTHF) Emavundleni (Cape Town; South Africa) and Kenya Medical Research Institute (KEMRI; Kisumu; Kenya) ECHO trial sites. RNA-Seq was conducted on all samples (Supplementary Fig. 1). After quality filtering, paired RNA-Seq data from 152 participants (DMPA-IM, *n* = 59; LNG-implant, *n* = 45; Cu-IUD, *n* = 48) were considered suitable for downstream differential expression analysis (Supplementary Figs. 1, 2). Screening demographics, sexual and reproductive characteristics of the remaining subjects were similar between arms, with no differences for age, body mass index (BMI), geographic location, marital status, number of sexual partners and condom use (Table 1). There were also no differences in the overall FGT microbial communities and infections (including prevalence of bacterial vaginosis [BV; by Nugent score], yeast [hyphae by microscopy] or *Neisseria gonorrhoeae* infection [Table 1]), with the exception of *Chlamydia trachomatis*. Laboratory diagnosed *C. trachomatis* prevalence was significantly lower in women randomized to use LNG implant (0/45, 0.0%) compared to those using DMPA-IM (9/59, 15.3%) and Cu-IUD (10/48, 20.8%) (*P* = 0.007) (Table 1). Syndromic management was provided to symptomatic women at screening, and to asymptomatic women confirmed to have an STI by laboratory-testing. The differential expression analysis of the endocervical transcriptome in women who were *C. trachomatis* positive versus negative at screening, using DESeq2, showed only 23 differentially expressed genes (DEGs). This suggested that treatment for *C. trachomatis* infection did not markedly alter the transcriptome, and we thus conducted our downstream contraceptive analysis without adjusting for chlamydia/antibiotic treatments (Supplementary Table 1).

**Table 1:** Demographics and metadata of ECHO subjects recruited to substudy of mucosal transcriptomics.

	DMPA-IM	Cu-IUD	LNG implant	P value
	(n = 59)	(n = 48)	(n = 45)	
Age (years)*	24 (21–28)	23 (19–27)	24 (22–27)	0.686
Body mass index (BMI) (kg/m^2^)*	22 (25–30)	24 (21–29)	22 (20–29)	0.620

Genital microbial communities and infections [n (%)]				
Lactobacillus dominance^$^	28/58 (48.3)	24/47 (51.1)	21/45 (46.7)	0.912
Bacterial vaginosis				0.912
Positive	17 (28.8)	17 (35.4)	15 (33.3)	
Intermediate	6 (10.2)	5 (10.4)	6 (13.3)	
Negative	36 (61.0)	26 (54.2)	24 (53.3)	
*Chlamydia trachomatis*	9 (15.3)	10 (20.8)	0 (0.0)	**0.007**
*Neisseria gonorrhoeae*	2 (3.4)	2 (4.2)	1 (2.2)	1.000
Yeast	2 (3.4)	3 (6.3)	4 (8.9)	0.519

Demographics [n (%)]				
Location				0.779
Emavundleni, South Africa	23 (40.0)	16 (33.3)	12 (26.7)	
KEMRI, Kenya	19 (32.2)	17 (35.4)	18 (40.0)	
Wits RHI, South Africa	17 (28.8)	15 (31.3)	15 (33.3)	
Marital status				0.140
Married	18 (30.5)	14 (29.2)	21 (46.7)	
Education				0.457
Primary school education	14 (23.7)	15 (31.3)	8 (17.8)	
Secondary education	43 (72.9)	30 (62.5)	33 (73.3)	
Post-secondary education	2 (3.4)	3 (6.3)	4 (8.9)	

Reproductive history				
Prior pregnancy	52 (88.1)	41	40 (88.9)	0.864
Currently breastfeeding	20/52 (38.5)	22/41 (53.7)	22/40 (55.0)	0.201

Sexual risk behaviour in the last 3 months [n (%)]				
>1 partner	6 (10.2)	6 (12.5)	1 (2.2)	0.164
New partner	6 (10.2)	3 (6.3)	1 (2.2)	0.275
Condom use				0.420
Always	15/57 (26.3)	9/44 (20.5)	16 (36.4)	
Sometimes	27/57 (47.4)	19/44 (43.2)	18 (40.9)	
Never	15/57 (26.3)	16/44 (36.4)	10 (22.7)	

BMI; body mass index, KEMRI; Kenya Medical Research Institute (Kisumu; Kenya), sd, standard deviation, Wits RHI; (Johannesburg; South Africa). *Median and interquartile range. ^$^Based on proteomics data.

A retrospective power analysis of each study arm helped to demonstrate that for majority of transcripts detected at a reasonable level above background (>10 reads counts), at *n* = 40 there was an estimated sensitivity to detect 96% of DEGs for DMPA-IM arm and 97% sensitivity for LNG and Cu-IUD study arms, at an absolute two-fold fold-change (Supplementary Fig. 3). These sensitivity statistics show that majority of DEGs were successfully detected through our analysis.

### 3.2 Cu-IUD, LNG implant and DMPA-IM initiation induce distinct patterns of endocervical gene-expression

**Fig. 1:**
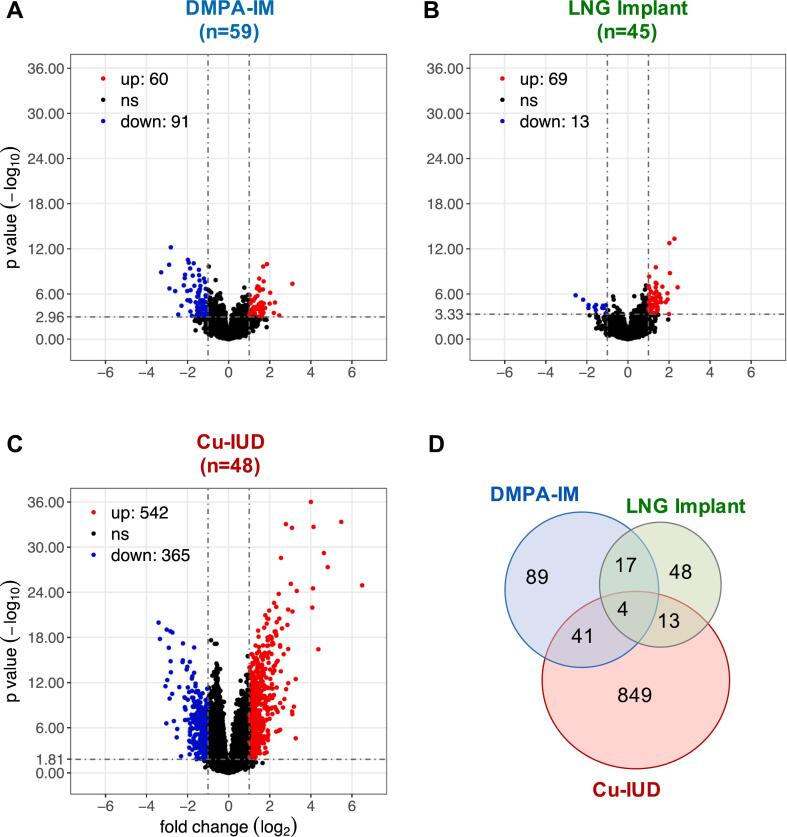
DMPA-IM, LNG implant and Cu-IUD initiation induce distinct patterns of endocervical gene-expression. The results of the statistical analyses on differential expression of post-vs-pre comparisons, i.e. DMPA-IM vs. baseline, LNG implant vs. baseline, and Cu-IUD vs. baseline, have been shown by the different panels (DMPA-IM: *n* = 59, LNG Implant: *n* = 45, Cu-IUD: *n* = 48). The significantly differentially expressed genes (DEGs) were identified by the following criteria: false discovery rate (FDR) <0.05, absolute fold-change >2, standard-error in fold-change (lfcSE) <1 and mean expression of gene >10. (A-C) Volcano plots showing statistical significance against fold-change for each comparison, with the DEGs highlighted in red (upregulated) and blue (downregulated) colors indicated in the upper quadrants. (D) Venn diagram showing the numbers of overlaps of DEGs across the three longitudinal comparisons. (For interpretation of the references to color in this figure legend, the reader is referred to the web version of this article.)

To assess the impact of initiation of contraception on the endocervical transcriptome, we identified DEGs induced after one month of contraception usage relative to baseline within each study arm in a longitudinal intention-to-treat (ITT) analysis (Supplementary Table 2). A total of 151 DEGs were induced by DMPA-IM (60 upregulated, 91 downregulated), 82 by LNG-implant (69 upregulated, 13 downregulated) and 907 DEGs by Cu-IUD initiation (542 upregulated, 365 downregulated) (Fig. 1). Along with the strikingly high number of DEGs, the Cu-IUD arm also demonstrated the highest degree of separation in the principal component analysis (PCA), indicating a much stronger impact of Cu-IUD on the endocervical gene-expression compared to the HCs (Supplementary Fig. 4). There was a limited overlap between DEGs identified in each study arm (Fig. 1D), indicating that initiation of each of the three contraceptives had a unique impact on host gene expression. Lastly, we compared our list of DEGs induced post one month of DMPA-IM initiation to data from a recently published observational study examining long-term (>6 months) DMPA administration [62]. Several DEGs were common between our data and those published by Bradley F. et al. (2022)^41^, including upregulation of GABRP, MMP12 and MMP13; and downregulation of SPRR2g, LCE3A and TGM3 (Supplementary Table 2).

### 3.3 DMPA-IM initiation induced an enrichment of T cell gene signatures in the FGT

**Fig. 2:**
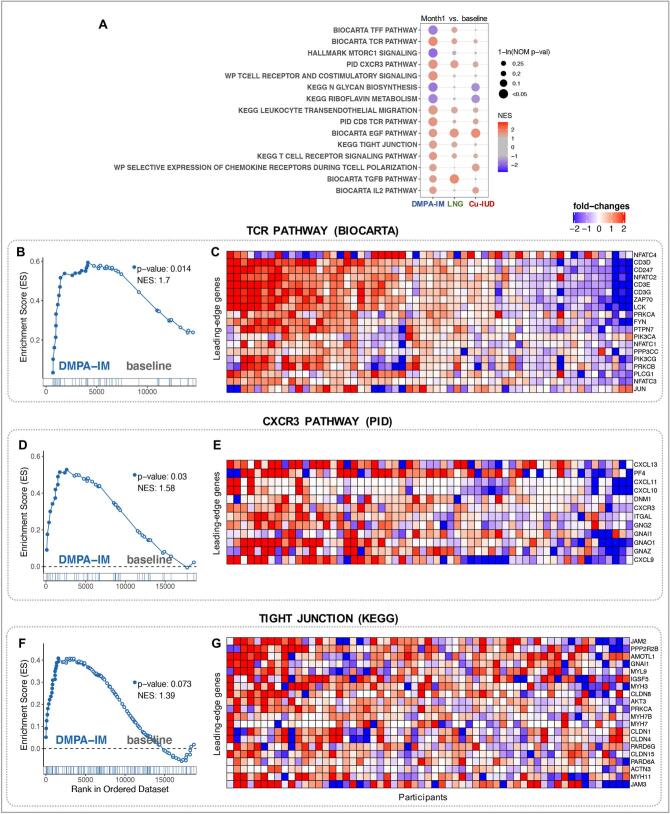
DMPA-IM initiation induced an enrichment of T cell gene signatures in the cervicovaginal tract (nominal *p*-value < 0.1). Important pathways enriched in DMPA-IM study arm, either at baseline or at month 1, were selected. (A) A dot plot to represent the gene set enrichment analysis (GSEA) statistics for these pathways, across the longitudinal comparisons in the three study arms. The statistical significance of the enrichment (1-ln (nominal p-value)) is shown by the size of the dots and the normalized enrichment score (NES) is represented by a blue-to-red color-gradient, blue for negative scores and red for positive scores. (B-G) Breakout enrichment line plots and leading-edge gene heatmaps for a few of these enriched pathways. (B,D,F) In the line plots, the running enrichment score (y-axis) is indicated for each gene ordered by their rank in the whole data set for that specific comparison, shown by the vertical bars below the x-axis. (C,E,G) Heatmaps for the leading-edge genes of the indicated pathways. The gene-expressions are log-transformed, further normalized by mean of baseline samples in the DMPA-IM. The color gradient goes from blue to red color in representing the lowest to the highest gene-expression across all samples in the comparison. (For interpretation of the references to color in this figure legend, the reader is referred to the web version of this article.)

The use of DMPA-IM has been reported to induce a diverse array of biological effects in the FGT. A closer examination of the 151 DEGs identified in DMPA-IM arm (Supplementary Table 2) showed significant alterations in a handful of genes that function in T cell activation (PRF1, TNFSF18, MAF) and tissue recruitment of immune cells (CXCL5, VCAM1). As a more sensitive approach to identify enriched gene pathways associated with contraceptive initiation, we performed Gene Set Enrichment Analysis (GSEA) using various well-defined gene-set/pathway collection databases from the Molecular Signatures Database (MSigDB) (https://www.gsea-msigdb.org/gsea/msigdb) [[44], [45], [46], [47], [48], [49]] and custom in-house gene-sets [32,[50], [51], [52], [53], [54]] (Supplementary Table 3) for each study arm comparing data from one month post-contraceptive initiation to baseline (Supplementary Table 4, Supplementary Fig. 5). Within the DMPA-IM arm, we observed an upregulation and enrichment of several gene sets associated with T cell activation, migration and communication, (e.g., T-cell receptor [TCR] and T-cell associated chemokine [CXCR3] signaling, interleukin [IL]-2) (Fig. 2). Within the CXCR3 pathway, the chemokines CXCL9, CXCL10, CXCL11 and CXCL13, that recruit activated T cells, and CXCR3 itself, were significantly enriched. Within the TCR pathways, genes expressing several subunits of the CD3 TCR complex were significantly elevated (Figs. 2B-E). Also, a significant enrichment of genes regulating tight junctions in epithelial barriers including several claudins (CLDN1, CLDN4, CLDN8, CLDN15) and junctional adhesion molecules (JAM2, JAM3) was observed (Figs. 2F, G). In contrast, gene pathways associated with growth and metabolism (e.g., glycolysis, fatty acid and protein metabolism, MTOR signaling) were downregulated in DMPA-IM (Fig. 2A). Lastly, DMPA use in NHPs has been reported to downregulate expression of interferon-stimulated genes (ISGs) [32]. In the current study, even though the expression of several ISGs (Supplementary Table 2) and cumulative enrichment of ISGs (IFN alpha response, Supplementary Table 4) were lower post-DMPA-IM usage, these trends were not significant.

### 3.4 DMPA-IM induced transcriptomic alterations are not strongly driven by microbial composition

We performed a sub-analysis to investigate whether the transcriptomic changes observed within the DMPA-IM arm were specific to the vaginal microbiota of participating women, i.e. *Lactobacillus*-dominated (LD, *n* = 25) or *Lactobacillus*-depleted (non-LD, *n* = 22) vaginal microbiota at the time of contraceptive initiation (as determined by 16S rRNA sequencing [55]) (Supplementary Table 5). Differential gene expression analysis of the dataset consisting of both LD and non-LD samples identified 114 DEGs (63 upregulated, 51 downregulated) while the datasets with only LD and only non-LD samples showed 36 and 24 DEGs, respectively (Supplementary Table 5, Supplementary Figs. 6A-C). A small overlap between the DEGs in the LD and non-LD groups was observed (Supplementary Fig. 6D). This suggests that DMPA-IM induced changes to the endocervical transcriptome were not markedly dependent on the baseline bacterial community but may be influenced by it. Several pathways were found to be similarly upregulated or downregulated within the combined LD + non-LD, LD only and non-LD only groups (Supplementary Fig. 6E-G, Supplementary Table 6). However, pathways associated with regulation of lymphocyte activation (NFAT Pathway, NK Cells Pathways, WNT Beta Catenin, MTOR, IgA Production) were specifically enriched in the LD subset. Conversely, several metabolic pathways (Fructose/Mannose, Amino Acid & Nucleotide Sugar, Pantothenate) were uniquely downregulated in the non-LD group (Supplementary Fig. 6E-G, Supplementary Table 6). Collectively, these results indicate that while there are signaling pathways that are unique to women based on their cervicovaginal microbiome status, its overall contribution to DMPA-induced gene expression is modest.

### 3.5 Growth factor signaling and epithelial barrier pathways enriched in women assigned to LNG implant

**Fig. 3:**
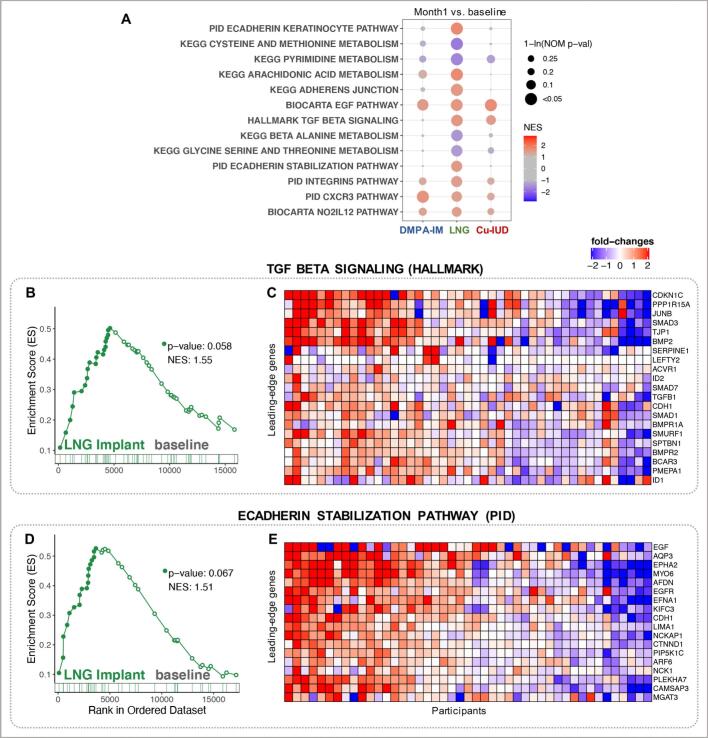
Growth factor signaling and epithelial barrier pathways enriched in women assigned to LNG implant *(*nominal p-value < 0.1). Important pathways enriched in LNG Implant study arm, either at baseline or at month 1, were selected. (A) A dot plot to represent the gene set enrichment analysis (GSEA) statistics for these pathways, across the longitudinal comparisons in the three study arms. The statistical significance of the enrichment (1-ln (nominal p-value)) is shown by the size of the dots and the normalized enrichment score (NES) is represented by a blue-to-red color-gradient, blue for negative scores and red for positive scores. (B-E) Breakout enrichment line plots and leading-edge gene heatmaps for a few of these enriched pathways. (B,D) In the line plots, the running enrichment score (y-axis) is indicated for each gene ordered by their rank in the whole data set for that specific comparison, shown by the vertical bars below the x-axis. (C,E) Heatmaps for the leading-edge genes of the indicated pathways. The gene-expressions are log-transformed, further normalized by mean of baseline samples in the LNG Implant. The color gradient goes from blue to red color in representing the lowest to the highest gene-expression across all samples in the comparison. (For interpretation of the references to color in this figure legend, the reader is referred to the web version of this article.)

Within the LNG implant arm, several pathways associated with cell proliferation and growth including growth factor signaling by epidermal growth factor (EGF) and transforming growth factor (TGF)-β, Notch signaling, and WNT signaling, as well as cell-cell adhesion pathways were positively enriched, whereas pathways associated with cell cycle control and DNA repair were downregulated (Fig. 3, Supplementary Fig. 5). Similar to that in DMPA-IM arm, we observed significant enrichment of genes associated with CXCR3 signaling (Fig. 3A) in LNG implant arm, however no concurrent signatures of enriched T cells were detected.

### 3.6 Cu-IUD usage causes significant perturbations to the cervicovaginal transcriptome

**Fig. 4:**
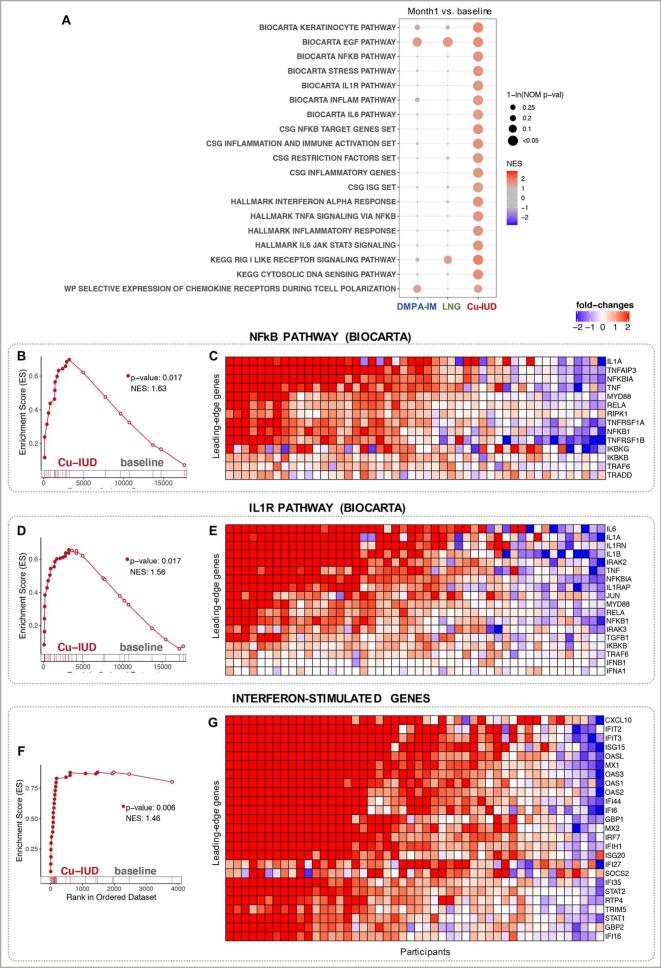
Immune activation pathways enriched in women assigned to Cu-IUD (nominal p-value < 0.1). Important pathways enriched in Cu-IUD study arm, were selected. (A) A dot plot to represent the gene set enrichment analysis (GSEA) statistics for these pathways, across the longitudinal comparisons in the three study arms. The statistical significance of the enrichment (1-ln (nominal p-value)) is shown by the size of the dots and the normalized enrichment score (NES) is represented by a blue-to-red color-gradient, blue for negative scores and red for positive scores. (B-G) Breakout enrichment line plots and leading-edge gene heatmaps for a few of these enriched pathways. (B,D,F) In the line plots, the running enrichment score (y-axis) is indicated for each gene ordered by their rank in the whole data set for that specific comparison, shown by the vertical bars below the x-axis. (C,E,G) Heatmaps for the leading-edge genes of the indicated pathways. The gene-expressions are log-transformed, further normalized by mean of baseline samples in the Cu-IUD. The color gradient goes from blue to red color in representing the lowest to the highest gene-expression across all samples in the comparison. (For interpretation of the references to color in this figure legend, the reader is referred to the web version of this article.)

**Fig. 5:**
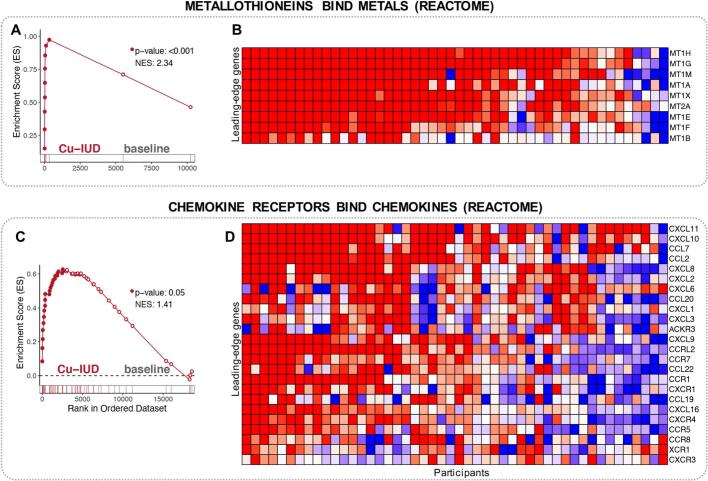
Metallothioneins and Chemokines pathways' enriched in women assigned to Cu-IUD (nominal p-value < 0.1). (A-D) Breakout enrichment line plots and leading-edge gene heatmaps for the pathways shown in the fig. (A,C) In the line plots, the running enrichment score (y-axis) is indicated for each gene ordered by their rank in the whole data set for that specific comparison, shown by the vertical bars below the x-axis. (B,D) Heatmaps for the leading-edge genes of the indicated pathways. The gene-expressions are log-transformed, further normalized by mean of baseline samples in the Cu-IUD. The color gradient goes from blue to red color in representing the lowest to the highest gene-expression across all samples in the comparison. (For interpretation of the references to color in this figure legend, the reader is referred to the web version of this article.)

Cu-IUD treatment, with 907 DEGs identified, had a dramatic impact on gene expression (Fig. 1C). Multiple pathways comprised of genes driving cytokine regulated inflammation were enriched (Fig. 4A). Specifically, genes in the NFKB, stress response, IL1 signaling, IL6 signaling and TNF signaling pathways were significantly enriched (Fig. 4A). Visualization of the leading-edge genes in the NFKB and IL1R pathways demonstrated that inflammatory gene expression was strongly and consistently upregulated in the majority of participants. Several inflammatory cytokine genes were robustly and significantly upregulated, including IL1A, IL1B, TNF, IL6, IL15, IL3A, IL12B and IL122 (Fig. 4C, E; Supplementary Table 2). Multiple chemokine genes regulating the recruitment of activated macrophages, T cells and neutrophils (CCL7, CCL8, CCL20, CCL22, CXCL1, CXCL2, CXCL6, CXCL8, CXCL9, CXCL9, CXCL10, CXCL11) were significantly upregulated (Fig. 5B).

We also observed highly significant enrichment of gene sets comprising ISGs and innate immune signaling (Fig. 4A, F, G). Transcripts encoding ISGs were among the highest upregulated genes in terms of both read count and fold-change relative to baseline. The upregulation of interferon-related pathways wa s unique to the participants in the Cu-IUD arm. Unlike the inflammatory pathways, in which the upstream cytokine genes were robustly induced, we did not observe significant upregulation of either IFNA or IFNB transcripts (Supplementary Table 2), albeit both of these genes IFNA and IFNB were detected within the leading-edge genes of the IL1R pathway (Fig. 4C). Given the magnitude of the ISG response, we examined genes known to be uniquely or highly expressed on plasmacytoid dendritic cells (pDCs), which are potent producers of Type I Interferon. We observed significant upregulation of IL3RA/CD123 gene, a marker expressed on pDCs and basophils. Transcripts for LILRA4/ILT7 and CLEC4C/BDCA2, both uniquely expressed by pDCs, trended towards increased expression after Cu-IUD initiation (Supplementary Table 2). Similarly, the transcription factors interferon-regulatory factor 7 (IRF7) and IRF8, also expressed by pDCs, were significantly induced (Supplementary Table 2).

Lastly, we observed widespread induction of genes in the metallothionein (MT) family in mucosal samples post Cu-IUD initiation, including eight isoforms of MT1 genes (MT1A, MT1B, MT1H, MT1M, MT1G, MT1X, MT1E, MT1F). MT1A and MT1B had the highest fold-change of all DEGs detected, at >1000-fold and 60-fold, respectively. MT2A was also significantly elevated. Similarly, genesets including metallothionein and metal stress responses were determined to be significantly enriched (Fig. 5, Supplementary Fig. 5). Collectively, these results indicate that Cu-IUD initiation induced a widespread inflammatory response, easily detectable at one-month post-initiation. These data also demonstrate that Cu-IUD initiation leads to an extensive induction of the type I IFN system, concurrent with elevated detection of molecules unique to pDCs and, initiation of the metallothionein system within the FGT.

### 3.7 Cu-IUD initiation recruits innate immune cells into the endocervix

**Fig. 6:**
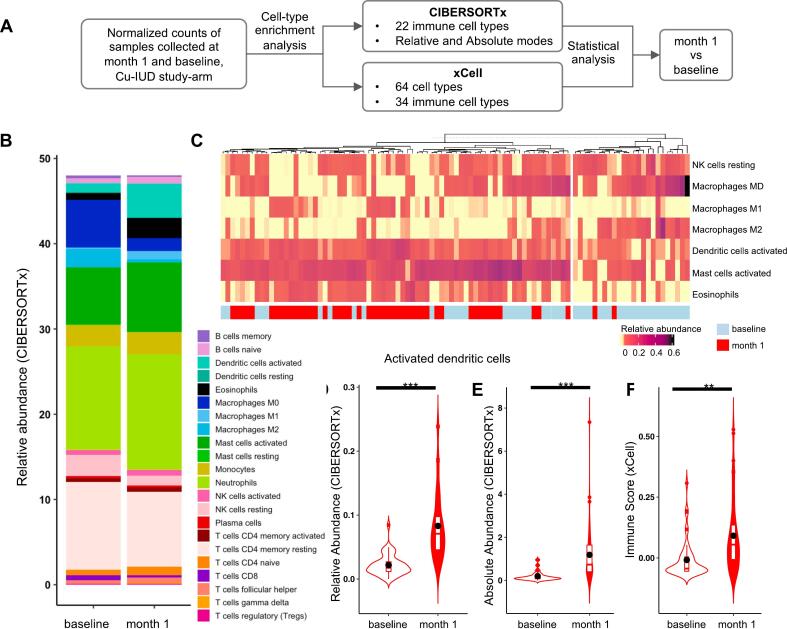
Cu-IUD initiation induces significant changes in the elements of innate immunity in endocervical tissue. (A) Workflow of in silico flow cytometry on RNA-seq data of women assigned to Cu-IUD. (B) Relative abundance of immune cell types in baseline and one month Cu-IUD samples using CIBERSORTx algorithm. (C) Hierarchical followed by k-means clustering of Cu-IUD samples according to the top significantly differentially abundant immune cell types. (D—F) The violin plots show the proportion of activated dendritic cells in samples collected at baseline and at one month, computed based on xCell and CIBERSORTx deconvolution algorithms. The two proportions were compared using Wilcoxon–Mann–Whitney test. ** and *** represents q-values ≤0.01 and 0.001, respectively.

Since Cu-IUD initiation resulted in the highest significant changes in the FGT gene expression as compared to LNG implant and DMPA-IM, its influence on the immune cell populations was further investigated using digital flow cytometry methods (Fig. 6A). For each immune cell type, its average abundance and the difference between post-Cu-IUD initiation and baseline was computed using two deconvolution methods (Supplementary Table 7). Neutrophils, T cells, CD4^+^ memory resting and activated mast cells were among the most abundant immune cell types in the Cu-IUD arm at one month post-contraception-initiation (Fig. 6B). Significant changes in the population of immune cells, particularly in cells regulating innate immunity were noted (Fig. 6C). Different deconvolution algorithms consistently detected a significant increase in the proportion of activated dendritic cells (Fig. 6D-F). Gene signatures representative of pDCs were determined to be significantly upregulated (Supplementary Table 7), which was consistent with the detection of pDC marker genes and transcription factors from the traditional RNA-Seq analysis. Collectively, these gene signature analyses indicat that Cu-IUD caused an enrichment of innate cells, particularly macrophages and pDCs, in the FGT.

### 3.8 Copper ions do not exert inflammatory effects on endocervical epithelial cells

**Fig. 7:**
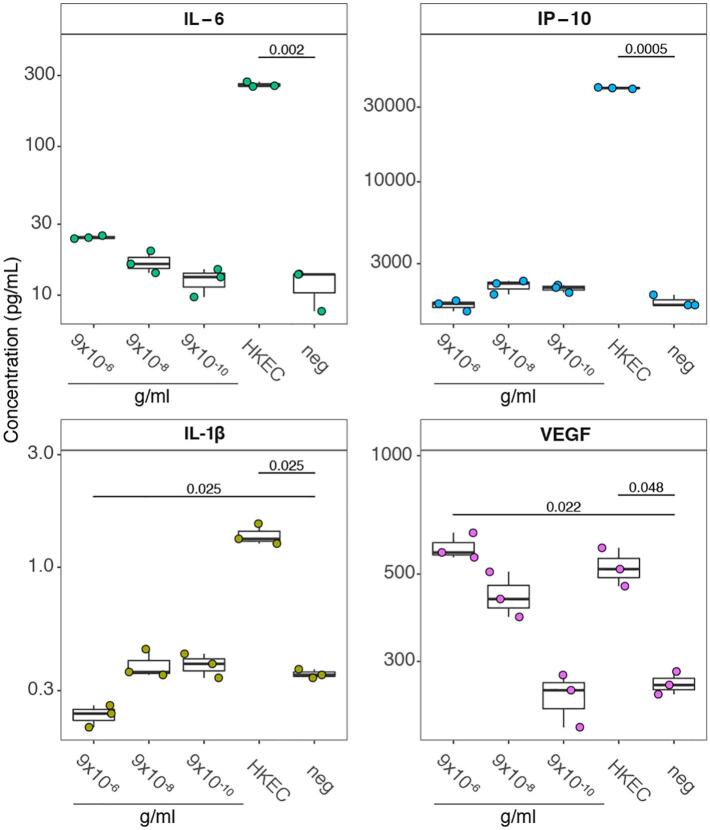
The concentrations of multiple endocervical cytokines are altered by biologically relevant copper (Cu^2+^) concentrations. Boxplots display the median and interquartile range. *P* values were determined using a Student's *t*-test and were corrected for multiple comparisons. Only values that remained significant after correction are displayed.

To test if the inflammatory events observed after Cu-IUD initiation in vivo were due to a direct effect of Cu^2+^ ions on cervical endothelia, we utilized a culture system of human endocervical epithelial cells (END1/E6E7) incubated with decreasing concentrations of CuCl_2_. END1/E6E7 cells exposed to heat-killed *E.coli* (positive control) elicited significantly elevated levels of IL-6, IP-10 (CXCL10) and IL-1b (Fig. 7). In contrast, incubation of END1/E6E7 with CuCl_2_ failed to elicit significant release of inflammatory cytokines at concentrations ranging from 9 × 10^−10^ to 9 × 10^−6^ g/ml, representing a range of phyiological concentrations of CuCl_2_ determined in the uterine fluids of women using T-380A IUD [63]; higher concentrations were found to be cyotoxic (data not shown). CuCl_2_ exposure induced elevated levels of VEGF protein (Fig. 7). This observation was consistent with the significantly upregulated expression of VEGFA transcripts, as well as other markers of oxidative stress, such as metallothioneins in the RNA-Seq data from Cu-IUD arm (Supplementary Table 2). Hence, these results indicate that the inflammatory signals observed in samples from Cu-IUD arm were due to Cu^2+^ ions induced angiogenesis, a known effect of Cu-IUD, and not due to a direct effect of these ions on the cervical epithelium.

## 4 Discussion

Although, sub-Saharan African women are at high risk for both STIs, including HIV, and unintended pregnancies, the impact of commonly used NBC methods on the mucosal immune environment has not been studied in detail using randomized designs. In this study, we examined the effects of three common and highly effective NBCs on endocervical gene expression of Kenyan and South African women enrolled into the ECHO trial, using high throughput transcriptomics.

We found that women assigned to Cu-IUD had dramatic endocervical transcriptional perturbations compared to women assigned to DMPA-IM and LNG implant and that these transcriptomic changes were associated with enrichment of immune activation pathways including inflammation and anti-viral responses. Accordingly, using data from the same ECHO cohort, we have previously reported that Cu-IUD induced significant increases in cytokine concentrations six months post-insertion compared to baseline [64], and that Cu-IUD initiation was associated with an increase in genital bacterial diversity and abundance of inflammatory bacteria, but not DMPA-IM and LNG implant [55]. Similar results have also been observed in other longitudinal observational cohorts [65,66]. Our results corroborate the mechanism of action of Cu-IUD as a contraceptive, i.e. the induction of a local inflammatory response, which inhibits sperm motility and migration, and ultimately fertilization. The potential for increased inflammation with Cu-IUD insertion could have profound consequences for women at high risk of STIs. Consistent with this, women randomized to the Cu-IUD arm in the larger ECHO trial had significantly higher *N. gonorrhea* prevalence at study exit compared to those in the DMPA-IM arm [67].

One of the more striking findings within the Cu-IUD arm, in addition to upregulation of inflammatory and interferon-related pathways, was the significantly upregulated MT genes,. The MT1 gene family has ten known functional genes [68], and we observed significant upregulation in eight MT1 members, up to 1000-fold that of pre-Cu-IUD insertion levels. MT1 proteins are responsible for regulating metal ions, and are thought to protect the host from heavy metal poisoning and oxidative stress [69]. The induction of MT1 expression has been reported for several metal ions, including Cu [68]. As the mechanism of action of Cu-IUD (TCu380A) is via elevated copper ions [70], our observation of elevated MT1 gene family is most likely a direct effect of the Cu itself. To our knowledge, this is the first study to report elevated levels of the MT1 family induced by contraception. In other recent findings, our group has observed that women receiving Cu-IUD insertion have perturbations in the vaginal microbiome, exhibiting decreases in the abundance of *Lactobacillus* species, increased BV, and increased bacterial loads [55], as well as elevated levels of inflammatory cytokines [64]. In our in vitro system, incubation of vaginal endothelial cells with Cu^2+^ did not elicit cytokine production directly. Taken together, suggests that the change in vaginal microbiota from the Cu-IUD is the mechanism through which the inflammatory transcriptional pathways we observed were induced in vivo. However, one limitation of our in vitro system is that it lacks immune cells, and thus we cannot rule out the possibility that Cu^2+^ exposure would be able to drive inflammation via immune cells resident in the FGT.

In this study, we found that DMPA-IM initiation was associated with an enrichment of gene pathways associated with T cell responses, consistent with an enrichment of infiltrating T cells into the cervicovaginal environment. Some observational studies have associated DMPA-IM with elevated levels of cervical immune cells, including CD4+ T-cells, and increased expression of the HIV co-receptor CCR5 on these cells [37,[71], [72], [73], [74], [75], [76], [77]] but other studies of DMPA employing a similar design have not observed increased T cell trafficking [7,65,78]. Using flow cytometry analysis of cervical cytobrushes from the ECHO trial, we previously reported no contraceptive-induced changes within or between contraceptive arms in the overall frequencies of CD4+ T cells or in the frequency of activated CD4+ T cells expressing CD38 or CCR5 [79]. However, we did observe that DMPA-IM initiation was associated with an increased proportion of endocervical Th17 cells including activated CD38+ Th17 cells compared to baseline [79]. This was not observed in Cu-IUD or LNG implant users. Together, these data suggest that DMPA-IM can alter the cervical Th17 population. Th17 cells have been identified as preferential target cells in vaginal transmission of HIV [80]. Despite this, the increase in Th17 cells observed in the DMPA-IM arm was not accompanied by a significantly increased HIV risk in the main ECHO trial compared with LNG-Implant or Cu-IUD arms. Th17 cells also play an important role in antibacterial and -fungal immunity at mucosal sites [[81], [82], [83]] and maintanence of the epithelial barrier [[84], [85], [86]]. Indeed, proteomic signatures of epithelial barrier repair were associated with higher frequencies of Th17 cells^68^. We did not identify transcriptomics signs of vaginal epithelial barrier disruption in women assigned to DMPA-IM in this study. This is in contrast to several previous observational studies [[20], [21], [22], [23], [24], [25],^28^,^29^], including a recent study by Zalenskaya et al., (2018) [26], in which whole-genome transcriptome profiling of the ectocervical mucosa of DMPA-IM users before and after 6 weeks of use was performed. Here, DMPA-IM use was found to result in downregulation of genes encoding cell junction proteins involved in epithelial integrity and differentiation [26]. Similarly, a more recent cross-sectional study of long-term DMPA-IM usage in Kenyan women reported a mild dowregulation of genes involved in epithelial structure [62]. The differences observed between these studies could reflect the timing of sampling and thus the MPA concentration [21,31], the specific compartment analyzed [76,87,88] or the experimental approach.

In our analysis of DMPA-treated women, we also considered if the vaginal microbiome was influencing the local transcriptome based on *Lactobacillus* status, as prior work had indicated that effects of the presence of non-*Lactobacillus*-dominant microbiome may mask the effects of DMPA [19]. However, after segregating DMPA-treated subjects based on *Lactobacillus*-dominance, we found that the vaginal transcriptomic changes induced by DMPA-IM were highly similar. This finding is in agreement with a recent report by Bradley and colleagues, who observed no interaction of the *Lactobacillus* status on DMPA-induced gene expression in the FGT of Kenyan women [62].

Published data on the influence of LNG implants on the FGT transcriptome is sparse. In this study, we found limited endocervical transcriptomic alterations in women randomized to the LNG implant arm. We did however observe an enrichment of pathways associated with cell proliferation and growth and a decrease in expression of pathways associated with cell cycle control and DNA repair. These transcriptomic changes differed from those observed in the women using DMPA-IM despite both contraceptives being progestin based, highlighting the differential impact of progestins with different pharmacokinetic and pharmacodynamic properties on the FGT milieu as have been shown in vitro [16,89,90].

## 5 Conclusions

These results illustrate the variable influence of different contraceptives on the FGT host environment with Cu-IUD insertion resulting in the highest perturbation of the endocervical transcriptome, mainly related to immune responses, as compared to DMPA-IM and LNG implant initiation. DMPA-IM use led to an enrichment of pathways associated with T cell responses and a reduction of pathways associated with growth and metabolism while LNG implant initiation was associated with enrichment of growth factor signaling.

The following are the supplementary data related to this article.

Table S1 DESeq results for Chlamydia-based analysis Table S1

Table S2 DESeq results for paired-analysis (post-vs-pre) for each study-arm in a longitudinal intention-to-treat (ITT) analysis. Table S2

Table S3 Custom in-house gene-sets used for GSEA. Table S3

Table S4 DESeq results for Lacto-dominance(LD)/non lacto-dominance (nonLD) at baseline, paired-analysis Table S4

Table S5 DESeq results for Lacto-dominance(LD)/non lacto-dominance (nonLD) at baseline, paired-analysis Table S5

Table S6 Differentially expressed pathways in the DMPA-IM group, pre-treatment vs 1 mos post-treatment, separated by women with Lactobacillus-dominated vaginal microbiome (LD), Lactobacillus-depleted vaginal microbiota (non-LD) or both groups analyzed together (LD + nonLD). Table S6

Table S7 Influence of Cu-IUD initiation on the population of vaginal immune cells Table S7

Supplementary material Supplementary Figure 1: A schematic representation of the RNA-Seq workflow. (A) The flowchart of RNA-Seq analysis outlines the experimental and data analysis steps carried out in the study. (B) The number of samples at collection timepoints and after quality-based filtering in each study arm.
Supplementary Figure 2: Quality assessment of the RNA-seq data. (A-K) Plots to identify selection thresholds for quality-based filtering of samples. The distributions of (A-C) mapping of reads, (D) the number of reads uniquely mapped to the host, (E) the median coefficient of variation (CV) of gene coverage, (F-H) RNA integrity number (RIN) scores and (I-K) relative log expression plot of normalized counts help to identify samples that are outliers with metrics lying in the extreme value ranges. The final selection thresholds were as follows: number of reads uniquely mapped to the host >5M, median coefficient of variation (CV) coverage ≤1.4, RIN score ≥2 and not NA, variance in relative log expression (RLE) plot within [-3.5, 3.5], and month3 and their corresponding baseline samples were removed. (L-O) RLE plots for the final set of samples in each study arm.
Supplementary Figure 3: Retrospective power analysis for each study arm. (A,C,E) Line plots for power vs. average normalized gene counts for DMPA-IM, LNG Implant and Cu-IUD. The stratified power is represented by each line for a certain sample size, stratified by the average counts of genes. Sample sizes varying from 5-50 are represented in different colors as shown in the legend in panel A. For a sample size greater than 40, the power is between 0.6 and 0.8 for genes with low counts (between 0 and 10) but improves significantly for genes with counts higher than 10 reads. (B,D,F) Marginal power-related results have been shown for each pair of sample sizes (SS1 and SS2), including marginal power, true discovery (TD), false discovery (FD), and false discovery cost (FDC, defined as the number of FD divided by the number of TD).
Supplementary Figure 4: Principal component analysis (PCA) on paired-data from each study arm. PCA plots shown for each study arm, (A) DMPA-IM, (B) LNG Implant and (C) Cu-IUD. In each of the three plots, circles represent baseline samples and solid triangles represent month 1 samples.
Supplementary Figure 5: Pathways enriched in at least one of the three study arms (DMPA-IM, LNG Implant and Cu-IUD) after 1 month of randomized contraceptive in post-vs-pre comparisons (nominal p-value <0.1). Dot plots to represent the statistical significance (nominal p-values) and normalized enrichment scores (NES) in gene set enrichment analysis (GSEA), for several MSigDB pathway collections have been shown. The statistical significance of the enriched pathways is shown by the size of the dots (1-ln (nominal p-value)), the larger the dot, the higher is the statistical significance. The normalized enrichment score (NES) is represented by a blue-to-red color-gradient, blue for negative scores and red for positive scores. The pathways are ordered by the nominal p-value of DMPA-IM vs. baseline contrast, with the most statistically significant pathway shown at the top.
Supplementary Figure 6: Analysis on DMPA-IM subsets of participants with *Lactobacillus*-dominant (LD) /non *Lactobacillus*-dominant (nonLD) at baseline. The results of the statistical analyses on differential expression of post-vs-pre comparison in DMPA-IM study arm, specifically for groups defined by their LD status: (i) LD + nonLD set, samples from those participants that were classified as either LD or nonLD at baseline (n=47), (ii) LD subset, samples from those participants that were classified as LD at baseline and (iii) nonLD subset, samples from those participants that were classified as nonLD at baseline. The significantly differentially expressed genes (DEGs) were identified by the following criteria: false discovery rate (FDR) <0.05, absolute fold-change >2, standard-error in fold-change (lfcSE) <1 and mean expression of gene >10. (A-C) Volcano plots showing statistical significance against fold-change for each set, with the DEGs highlighted in red (upregulated) and blue (downregulated) colors indicated in the upper quadrants. (D) Venn diagram showing the numbers of overlaps of DEGs across the three sets. (E-G) Pathways enriched in at least on of the three sets (LD+nonLD, LD or nonLD subsets in DMPA-IM study arm) after 1 month of randomized contraceptive (nominal p-value <0.1). Dot plots to represent the statistical significance (nominal p-values) and normalized enrichment scores (NES) in gene set enrichment analysis (GSEA), for several MSigDB pathway collections have been shown. The statistical significance of the enriched pathways is shown by the size of the dots (1-ln (nominal p-value)), the larger the dot, the higher is the statistical significance. The normalized enrichment score (NES) is represented by a blue-to-red color-gradient, blue for negative scores and red for positive scores. The pathways are ordered by the nominal p-value of LD subset, with the most statistically significant pathway shown at the top. Supplementary material

## Funding

This work and the Evidence for Contraceptive Options and HIV Outcomes (ECHO) Study were made possible by the combined generous support of the Bill & Melinda Gates Foundation (Grant OPP1032115), the American people through the United States Agency for International Development (Grant AID-OAA-A-15–00045), the Swedish International Development Cooperation Agency (Grant 2017/762965–0), the South Africa Medical Research Council, and the United Nations Population Fund. Contraceptive supplies were donated by the Government of South Africa and US Agency for International Development. Funding to support this ancillary study of biological mechanisms was from the US National Institute of Child Health and Human Development R01 HD089831 to RH and HBJ. CB was supported by bursary funds from the 10.13039/501100001323Poliomyelitis Research Foundation (fund number 17/43) during her Ph.D. work. RNA Sequencing was conducted in the ENPRC Genomics Core which receives support from NIH grants P51OD011132 and S10OD026799. The content is the sole responsibility of the authors and does not necessarily represent the official views of the study funders.

## Data Availability

Transcriptomic data is available in the Gene Expression Omnibus (GEO) repository under accession number GSE190923. Custom R scripts and supporting documentation on the RNA-Seq analyses are available at https://github.com/BosingerLab/Gupta_etal_ECHO_RNASeq.
