# Supplementary material for: Systems analysis reveals differential expression of endocervical genes in African women randomized to DMPA-IM, LNG implant or cu-IUD
Source: Clin Immunol. 2023 Oct;255:109750. doi: 10.1016/j.clim.2023.109750 (PMC10570927; doi:10.1016/j.clim.2023.109750)
Supplement: Supplementary material — Supplementary Figure 1: A schematic representation of the RNA-Seq workflow. (A) The flowchart of RNA-Seq analysis outlines the experimental and data analysis steps carried out in the study. (B) The number of samples at collection timepoints and after quality-based filtering in each study arm. Supplementary Figure 2: Quality assessment of the RNA-seq data. (A-K) Plots to identify selection thresholds for quality-based filtering of samples. The distributions of (A-C) mapping of reads, (D) the number of reads uniquely mapped to the host, (E) the median coefficient of variation (CV) of gene coverage, (F-H) RNA integrity number (RIN) scores and (I-K) relative log expression plot of normalized counts help to identify samples that are outliers with metrics lying in the extreme value ranges. The final selection thresholds were as follows: number of reads uniquely mapped to the host >5M, median coefficient of variation (CV) coverage ≤1.4, RIN score ≥2 and not NA, variance in relative log expression (RLE) plot within [-3.5, 3.5], and month3 and their corresponding baseline samples were removed. (L-O) RLE plots for the final set of samples in each study arm. Supplementary Figure 3: Retrospective power analysis for each study arm. (A,C,E) Line plots for power vs. average normalized gene counts for DMPA-IM, LNG Implant and Cu-IUD. The stratified power is represented by each line for a certain sample size, stratified by the average counts of genes. Sample sizes varying from 5-50 are represented in different colors as shown in the legend in panel A. For a sample size greater than 40, the power is between 0.6 and 0.8 for genes with low counts (between 0 and 10) but improves significantly for genes with counts higher than 10 reads. (B,D,F) Marginal power-related results have been shown for each pair of sample sizes (SS1 and SS2), including marginal power, true discovery (TD), false discovery (FD), and false discovery cost (FDC, defined as the number of FD divided by the number of TD [file mmc8.pdf]

A

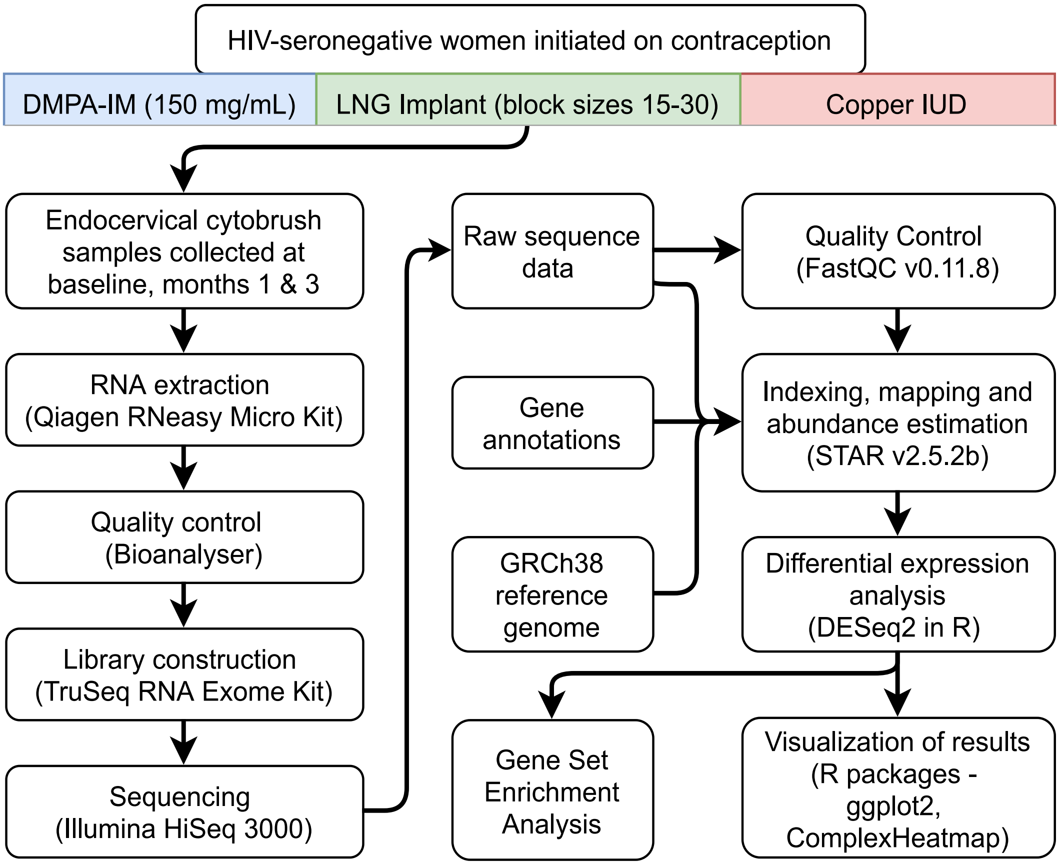

B

Study-arm	DMPA-IM	LNG Implant	Cu-IUD
Original set of samples (paired-data)	66 x 2	61 x 2	61 x 2
Filtered set of samples (paired-data)	59 x 2	45 x 2	48 x 2

Supplementary Figure 1

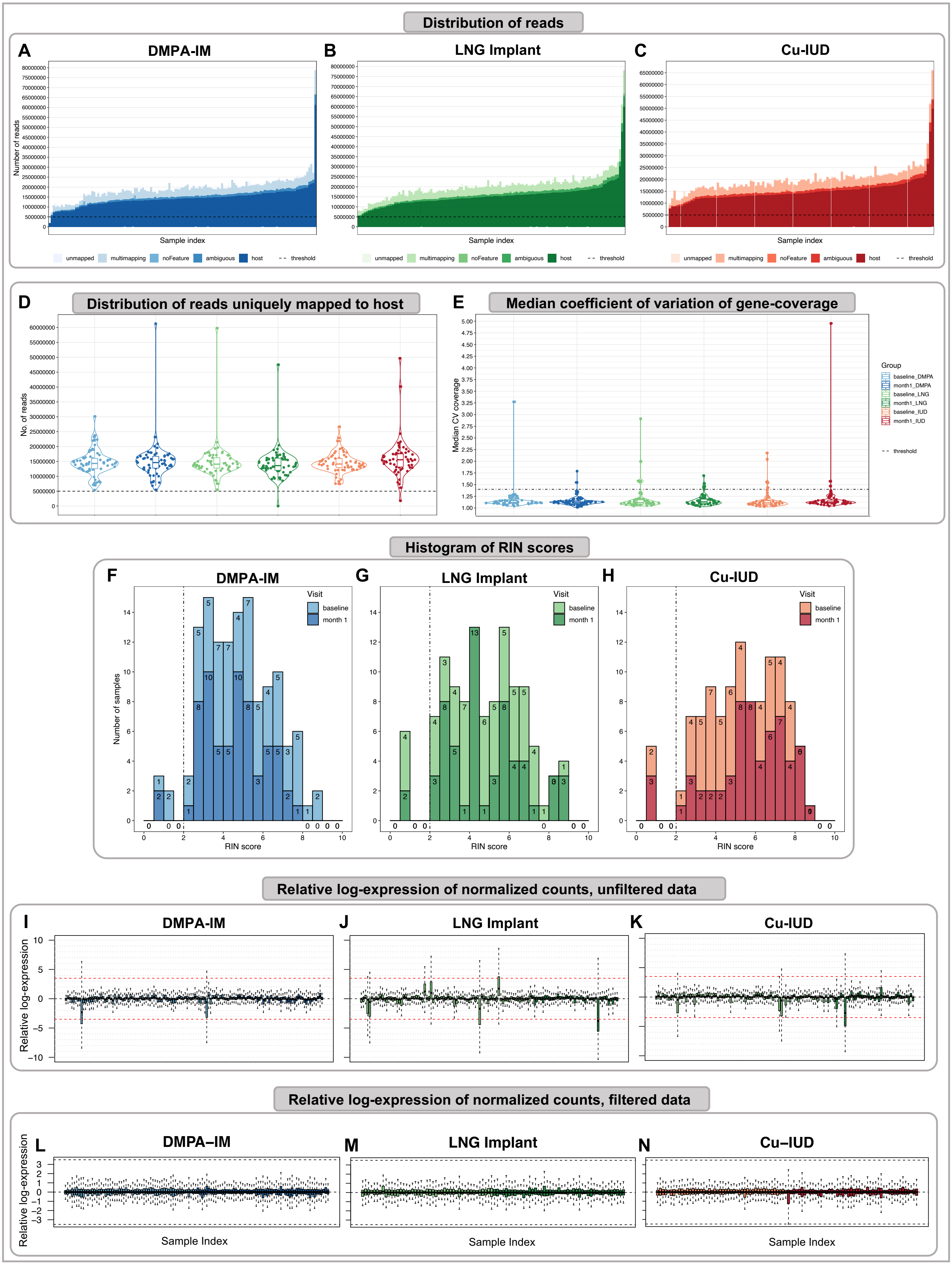

Supplementary Figure 2

DMPA-IM

A

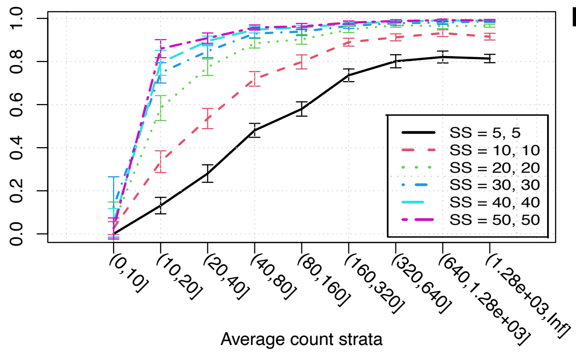

B

SS1	SS2	Nominal FDR	Actual FDR	Marginal power	Avg # of TD	Avg # of FD	FDC
5	5	0.05	0.28	0.64	222.75	100.65	0.45
10	10	0.05	0.17	0.81	281.10	74.85	0.27
20	20	0.05	0.11	0.91	314.75	55.40	0.18
30	30	0.05	0.09	0.94	327.40	45.60	0.14
40	40	0.05	0.08	0.96	332.90	43.95	0.13
50	50	0.05	0.07	0.97	335.60	41.75	0.12

LNG Implant

C

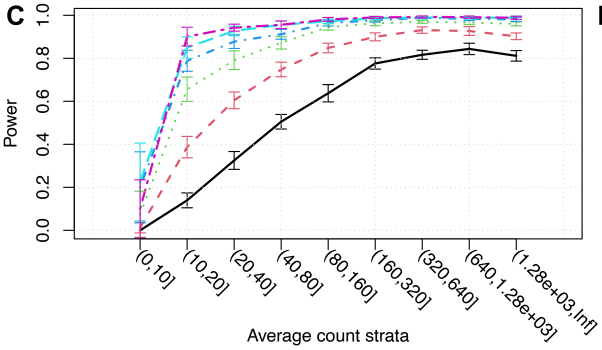

D

SS1	SS2	Nominal FDR	Actual FDR	Marginal power	Avg # of TD	Avg # of FD	FDC
5	5	0.05	0.28	0.68	221.35	100.25	0.45
10	10	0.05	0.17	0.84	273.00	70.25	0.26
20	20	0.05	0.10	0.92	301.65	50.00	0.17
30	30	0.05	0.08	0.95	312.05	42.35	0.14
40	40	0.05	0.07	0.97	317.80	36.45	0.12
50	50	0.05	0.07	0.98	319.80	38.35	0.12

Cu-IUD

E

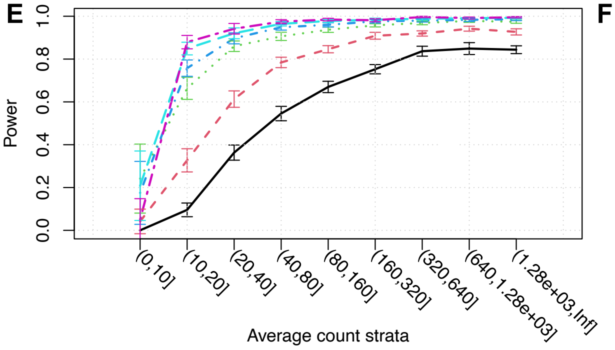

F

SS1	SS2	Nominal FDR	Actual FDR	Marginal power	Avg # of TD	Avg # of FD	FDC
5	5	0.05	0.24	0.69	279.50	101.25	0.36
10	10	0.05	0.14	0.84	339.90	72.40	0.21
20	20	0.05	0.10	0.93	377.25	54.80	0.15
30	30	0.05	0.07	0.96	386.95	43.60	0.11
40	40	0.05	0.07	0.97	392.95	43.45	0.11
50	50	0.05	0.06	0.98	396.00	39.70	0.10

Supplementary Figure 3

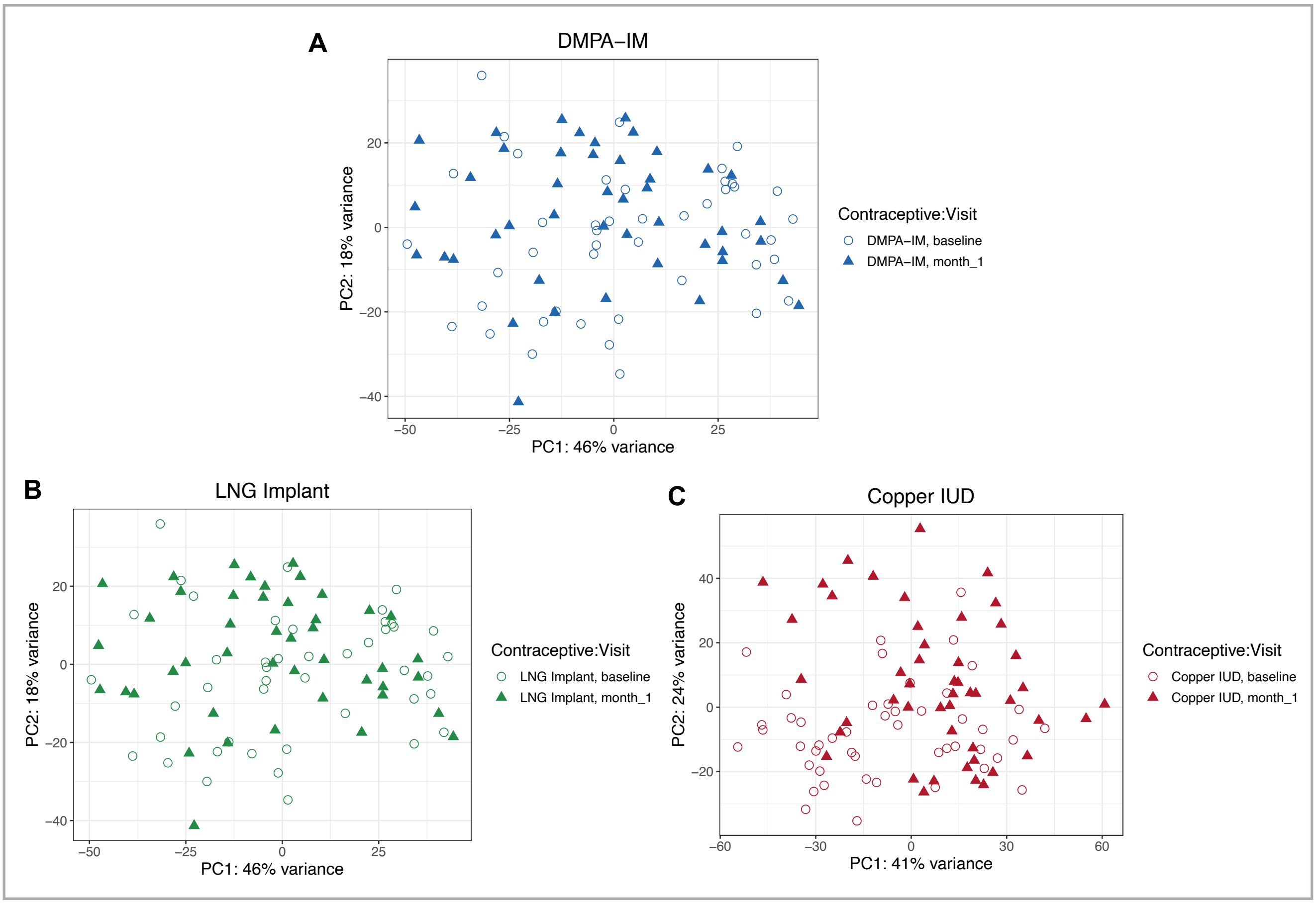

Supplementary Figure 4

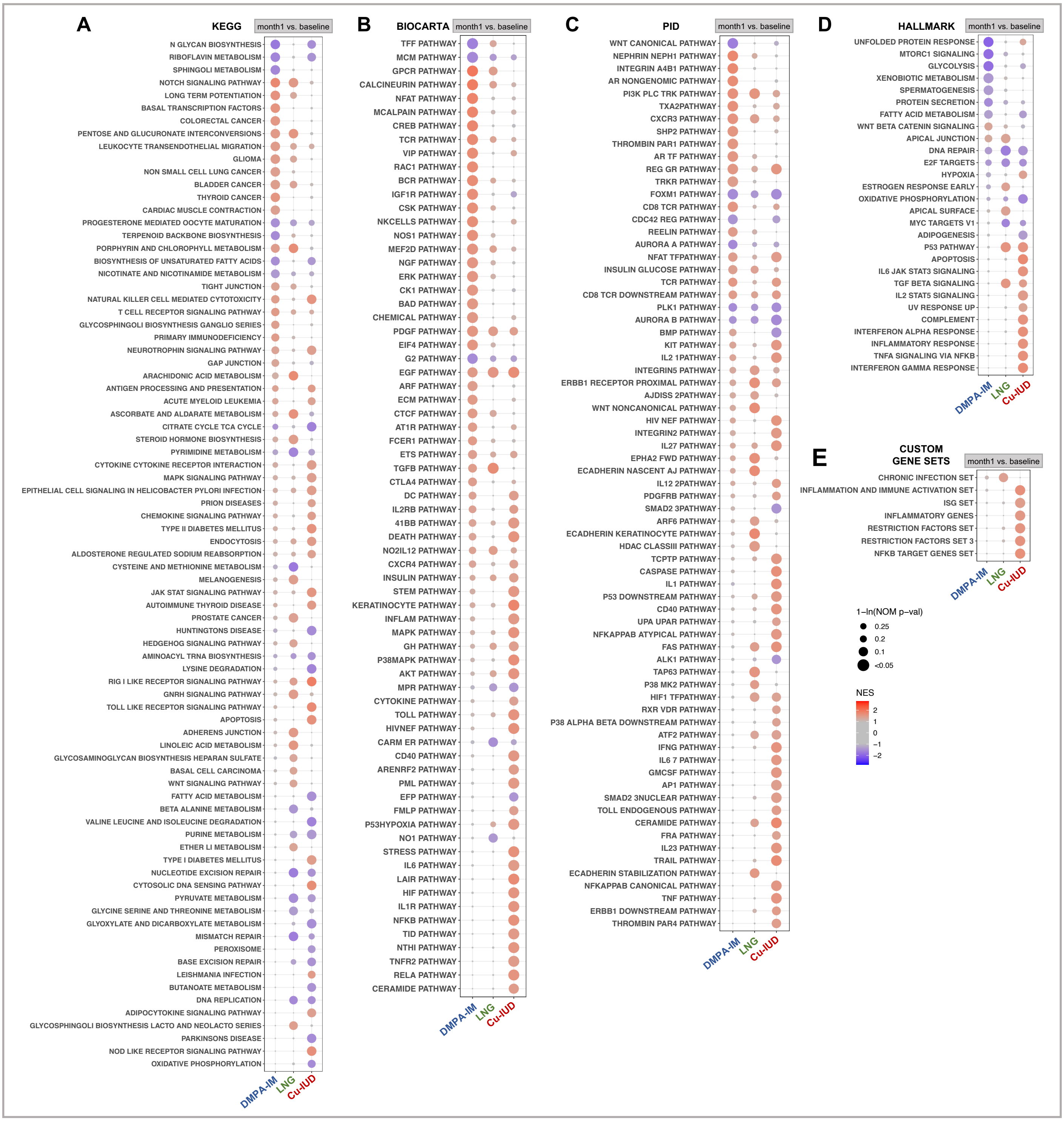

Supplementary Figure 5

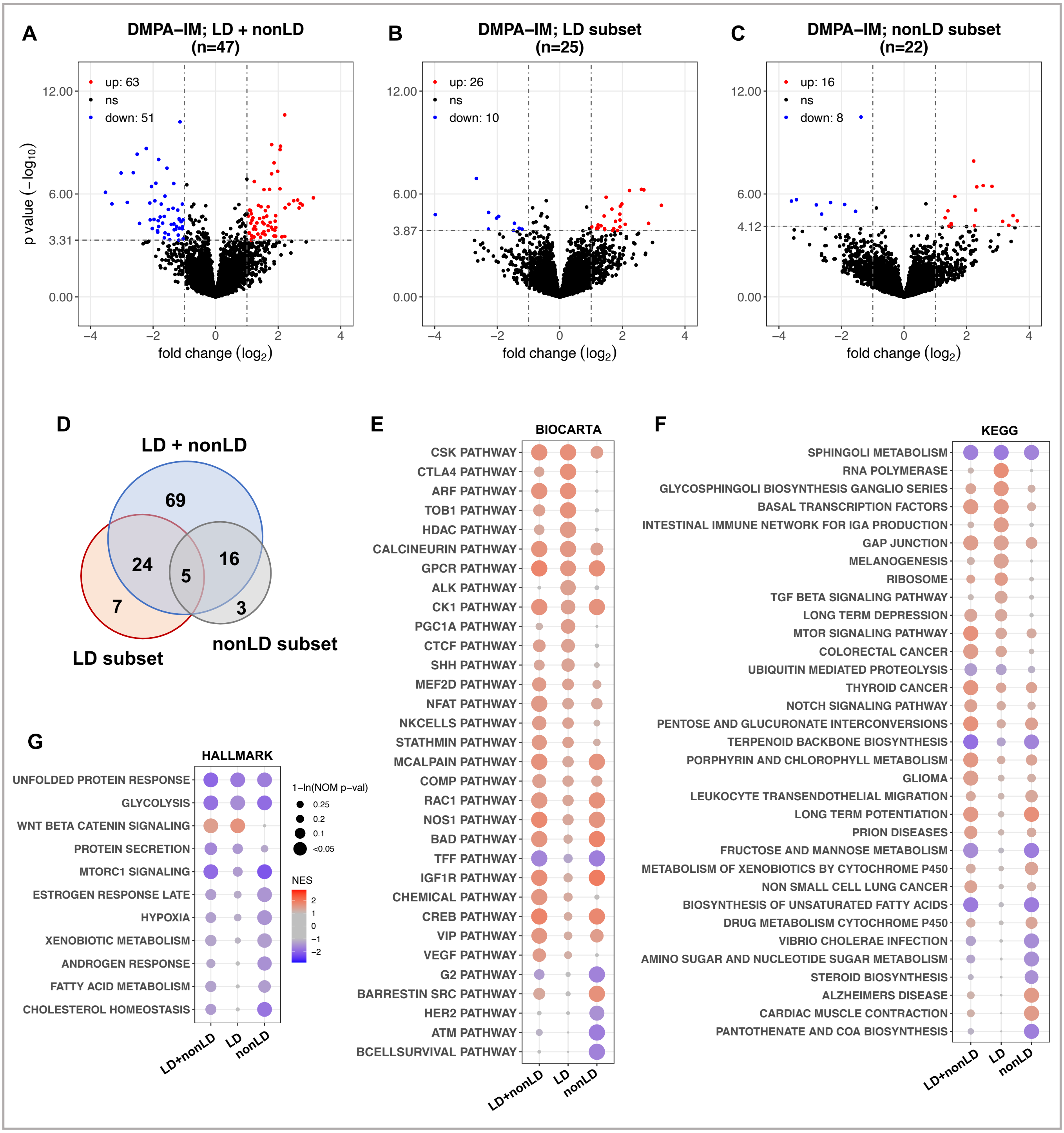

Supplementary Figure 6
